# LINC00355 promoted the progression of lung squamous cell carcinoma through regulating the miR-466/LYAR axis

**DOI:** 10.1590/1414-431X20209317

**Published:** 2020-10-21

**Authors:** XueFeng Sun, GuangSuo Wang, PeiKun Ding, ShiXuan Li

**Affiliations:** Department of Thoracic Surgery, Shenzhen People's Hospital, The Second Clinical Medical College of Jinan University, Shenzhen, Guangdong, China

**Keywords:** LINC00355, miR-466, LYAR, ceRNA, Lung squamous cell carcinoma

## Abstract

LINC00355 has been reported aberrantly over-expressed and associated with poor prognosis in various types of cancer. However, reports regarding the effect of LINC00355 on lung squamous cell carcinoma (SCC) are rare. This study aimed to explore the function of LINC00355 in the development and progression of lung SCC and reveal the underlying mechanism. The expression and subcellular location of LINC00355 were determined by qRT-PCR and RNA-FISH, respectively. The lung SCC cell growth was analyzed by CCK-8 assay, transwell invasion, wound healing, colony formation, and flow cytometry assays. Reactive oxygen species level was evaluated by DCFH-DA probes. Bioinformatics online websites, luciferase reporter assay, RNA binding protein immunoprecipitation (RIP), and RNA pull-down assays were utilized to investigate the interaction among LINC00355, miR-466, and Ly-1 antibody reactive clone (LYAR). The results showed that LINC00355 was upregulated in lung SCC and was positively associated with poor overall survival in lung SCC patients. LINC00355 was mainly located in the cytoplasm of SCC cells. Additionally, LINC0035 functioned as a competing endogenous RNA (ceRNA) to target miR-466, and LYAR was identified as a direct target of miR-466. LINC00355 expression negatively correlated with miR-466 level, and positively correlated with LYAR level. Mechanistically, knockdown of LINC00355 inhibited cell proliferation, migration and invasion, promoted cell apoptosis *in vitro*, and suppressed tumor growth *in vivo* through targeting miR-466, and thus down-regulated LYAR expression. These findings provide a new sight for understanding the molecular mechanism of lung SCC and indicate that LINC00355 may serve as a potential biomarker for the diagnosis and treatment of lung SCC.

## Introduction

Lung cancer is the world's leading cause of cancer-related deaths (1). More than 80% of lung cancers have been classified as non-small cell lung cancer (NSCLC) (2). Apart from adenocarcinoma, squamous cell carcinoma (SCC) is the most frequent subtype of NSCLC, representing approximately 30% of NSCLCs (3). Although the changes in tobacco consumption habits have decreased the incidence of SCC, effective treatment options to improve the outcome of lung SCC patients are still insufficient (4,5). For nearly two decades, platinum-based doublet chemotherapy is still considered as the standard first-line therapy for lung SCC (6). New treatments, mainly including bevacizumab, anaplastic lymphoma kinase (ALK) inhibitors, and EGFR tyrosine kinase inhibitors, have significantly improved the overall survival of patients with adenocarcinoma, but the therapeutic effects are not effective for lung SCC patients (7,8). Therefore, seeking new treatment methods are important for patients with lung SCC.

In recent years, many studies have shown that lung SCC is not only associated with cigarette smoking, but also related to genetic changes (9). In particular, long noncoding RNAs (lncRNAs) have attracted our attention due to their specific biological functions in various diseases (10). lncRNAs with more than 200 nt in length have gene-regulatory capacity, mainly including epigenetic regulation, imprinting, chromosome dosage-compensation, nuclear and cytoplasmic trafficking transcription, splicing, and translation (11). Emerging studies have suggested that the abnormal expression of lncRNAs plays critical roles in the pathogenesis of diverse types of cancer (12). For instance, lncRNA MALAT1 (metastasis-associated lung adenocarcinoma transcript 1) overexpression inhibits the metastasis of breast cancer through regulating the pro-metastatic transcription factor TEAD (13).

lncRNA LINC00355, also known as lnc-PCDH9-13:1, is a newly identified lncRNA. It has no protein-coding capacity, and can interact with chromatin and histone modifiers, thereby regulate gene expression (14). Several reports suggest that the abnormal expression of LINC00355 is linked to the overall survival of cancers such as prostate cancer, colorectal cancer, and colon adenocarcinoma (15–18). LINC00355 is regarded as a good candidate for understanding the mechanisms of sepsis (19). However, the effects and molecular mechanisms of LINC00355 on lung SCC have not been reported. Therefore, the effect of LINC00355 on lung SCC was determined by performing a series of biological experiments in this study.

## Material and Methods

### Bioinformatics method

The potential miRNAs that competitively bind with LINC00355 and the target gene of the candidate miRNA were investigated. Firstly, LNCipedia (https://lncipedia.org/) was used to search the sequences of LINC00355 and the transcript (LNCipedia transcript ID: LINC00355:1) was obtained. Next, the “Custom Prediction” column in miRDB website (http://mirdb.org/) was used to obtain the potential miRNAs. The top 15 miRNAs are listed in Supplementary Table S1. There are no reports on PubMed Central and publisher websites regarding miR-548j-3p, miR-548aq-3p, miR-548am-3p, miR-548ah-3p, miR-548ae-3p, miR-548x-3p, miR-548aj-3p, miR-4789-3p, miR-4758-5p, miR-1238-5p, and miR-3132. Only three reports regarding miR-1227-3p and miR-5193 were found, which provide little useful information for our study. Importantly, previous research showed that miR-466 could function as a tumor suppressor and was associated with cigarette smoke-related lung disease (20). MiR-494-3p as a carcinogenic factor was upregulated in lung cancer and promoted the progression of lung cancer (21). Based on the competing endogenous RNA (ceRNA) hypothesis and the fact that LINC00355 acts as a carcinogenic factor in several cancers (15–18), miR-466 was thus selected for the following analysis.

The target gene of miR-466 was identified by the Targetscan website (http://www.targetscan.org/vert_72/). The top 30 genes are listed in Supplementary Table S2, among which LYAR was selected for the following analysis.

### Clinical specimens

Thirty pairs of lung SCC specimens and the corresponding adjacent non-tumor tissues were collected from lung SCC patients without preoperative treatments (chemotherapy, radiotherapy, or other therapeutic methods) that underwent surgery at the Shenzhen People's Hospital from 2016 to 2018. Written informed consent was obtained from each patient. The present experiments received the approval from the Clinical Research Ethics Committee of Shenzhen People's Hospital (Approval No. 2019001). After resection, the tissues were obtained, frozen in liquid nitrogen, and stored at -80°C.

### Cell culture and transfection

Human lung squamous cell lines (SK-MES-1, NCI-H226, and A549) and human normal lung epithelial cell line (BEAS-2B) were obtained from Shanghai Institutes for Biological Science, China. In addition, human lung squamous cell line NCI-H2170 was obtained from ATCC (https://www.atcc.org/). All cell lines were cultured in DMEM medium (Gibco, USA) containing 10% fetal bovine serum (FBS) under 5% CO_2_ in a humidified incubator at 37°C.

shRNAs targeting LINC00355 (shLINC00355#1, shLINC00355#2, shLINC00355#3) and scrambled negative control shRNA (shNC) were synthesized and inserted into pcDNA3.1 (Invitrogen, USA). miR-466 inhibitor, inhibitor control (NC inhibitor), miR-466 mimic, and miRNA control mimic (NC mimic) were synthesized by Invitrogen. Transfection was performed by using Lipofectamine 3000 (Invitrogen).

### RNA extraction and quantitative real-time PCR (qRT-PCR)

Total RNAs from fresh-frozen tissues or cells were extracted using TRIzol reagent (GenMed, China). Using PrimeScript RT Reagent kit (Takara, China), total RNAs were reversely transcribed into cDNA. Using a TransGen One-Step qRT-PCR SuperMix kit (Changsha, China), qRT-PCR assays were used to detect messenger RNA. qRT-PCR was performed on the 7500 Fast Real-time PCR System (Applied Biosystems, USA). GAPDH was used as a reference gene. miRNA from fresh-frozen tissues or cells was extracted using Qiangen miRNeasy Mini kit (China). miRNA qRT-PCR was performed with miR-specific primers from the TaqMan miR assays (Applied Biosystems) on an Applied Biosystems StepOnePlus machine as per manufacturer's manual, and U6 was used as a reference gene. The sequence of primers are as follows: LINC00355 forward: 5′-ACA GAG CTG GTG GGA GCT GGG AAT-3′, reverse: 5′-AGT ATC AAT AGC TGA ATA GAC-3′; miR-466 forward: 5′-CAC TAG TGG TTC CGT TTA GTA G-3′, reverse: 5′-TTG TAG TCA CTA GGG CAC C-3′; (Ly-1 antibody reactive clone) forward: 5′-CCG TTG GCG TCA CTT CCA C-3′, reverse: 5′-AGC TAC CTG CCT CTC AGG CT-3′; U6 forward: 5′-CTC GCT TCG GCA GCA CA-3′, reverse: 5′-AAC GCT TCA CGA ATT TGC GT-3′; GAPDH forward: 5′-GGG AGC CAA AAG GGT CAT-3′, reverse: 5′-GAG TCC TTC CAC GAT ACC AA-3′.

### RNA fluorescence *in situ* hybridization (RNA-FISH)

RNA-FISH was performed to determine the subcellular location of LINC00355. In brief, NCI-H2170 cells were fixed in 4% formaldehyde for 15 min at room temperature, washed with PBS, and then permeabilized with 0.5% Triton X-100 on ice for 10 min. Next, cells were washed with PBS, rinsed in 2× saline sodium citrate (SSC), and dehydrated with the gradient concentration ethanol (70, 85, and 100%). Subsequently, cells were hybridized using the RNA probe-LINC00355 in hybridization buffer (2× SCC, 10% formamide, 100 mg/mL dextran sulfate) overnight at 42°C. After washing with hybridization buffer and 2× SCC, cells were incubated with DAPI (40,6-diamidino-2-phenylindole) at room temperature in the dark. Finally, LSM 800 confocal microscope (Carl Zeiss, Germany) was utilized to capture the images.

### Cell counting kit 8 (CCK-8) assay

After transfection, NCI-H2170 cells were seeded in 96-well plates (1.0×10^3^ cells/well) and incubated in DMEM medium containing 10% FBS for 12, 24, 48, and 72 h. Following the manufacturer's instructions, the CCK-8 detection kit (Engreen Biosystem Co, Ltd., China) was utilized to determine cell proliferation. The absorbance was measured at 450 nm by a microplate reader (Molecular Devices, USA).

### Colony formation assay

The transfected cells at a density of 600 cells per well were seeded in six-well plates, and cultured in DMEM medium containing 10% FBS in a humidified incubator with 5% CO_2_ at 37°C for 2 weeks, replacing the medium every 3 days. After washing twice with PBS, cells were fixed with methanol for 15 min at 25°C and then stained using 0.1% crystal violet. Finally, colony formation was counted manually.

### Flow cytometry

The transfected cells were harvested, washed three times with PBS, and re-suspended in binding buffer. Then, the samples were incubated with propidium (PI) and FITC-conjugated Annex V at 25°C for 15 min in the dark. Subsequently, cell apoptosis was analyzed by a flow cytometer (FACSCanto™ II, BD Biosciences, USA).

### Wound healing assay

After transfection, NCI-H2170 cells were collected, seeded in six-well plates, and grown in the complete culture medium to 90% confluence. In the cell monolayer, a scratch was made by a 200 μL pipet tip. After removing the scratched cells, the remaining adherent cells were cultured in serum-free DMEM medium for another 24 h. Then, the samples were photographed at 0 and 24 h under a microscope.

### Transwell invasion assay

The transfected cells were digested, suspended in serum-free DMEM medium, and seeded in Transwell chambers (Corning Costar, USA) coated with Matrigel (BD Biosciences). DMEM medium containing 10% FBS as a chemo-attractant was added into the lower chamber. On the upper surface of the chamber, the remaining cells were removed with a cotton swab after 24 h of incubation. On the lower surface of the chamber, the invaded cells were fixed with methanol, stained with 0.5% crystal violet solution, and photographed by microscope at five random fields.

### Western blot

Proteins from cells were extracted with RIPA lysis buffer (Beyotime Biotechnology, China) containing protease inhibitor cocktail. Protein lysates were centrifuged (at 16,000 *g* for 20 min at 4°C), collected, and the concentration was determined by BCA method. Proteins were equally separated on 12% SDS-PAGE, and transferred to polyvinylidene difluoride (PVDF) membrane (Millipore, USA). Next, the membranes were blocked for 1 h at room temperature by 5% non-fat milk, and incubated overnight at 4°C with primary antibodies (1:1000 dilution) against: PCNA (Cat No. 13110), E-cadherin (Cat No. 3195), N-cadherin (Cat No. 13116), Bcl-2 (Cat No. 4223), Bax (Cat No. 5023), cleaved caspase-3 (Cat No. 9661), LYAR (Cat No. ab233082), CHAC1 (Cat No. ab76386), and GAPDH (Cat No. 5174). After being washed with TBST three times, the membranes were incubated with secondary antibodies (Cat No. SA00001-2, 1:5000 dilution) at room temperature for 1 h, and then photographed using a chemiluminescence detection kit (Amersham Pharmacia Biotech, USA). The primary antibodies against PCNA, E-cadherin, N-cadherin, Bcl-2, Bax, Cleaved caspase-3, and GAPDH were purchased from Cell Signaling Technology (USA). The primary antibodies against LYAR and CHAC1 were obtained from Abcam (UK). The secondary antibodies (horseradish peroxidase (HRP)-conjugated goat anti-rabbit) were purchased from Proteintech Group (USA). The intensity of the protein bands was quantified by ImageJ software (NIH, USA).

### Luciferase reporter assay

The binding sites between LINC00355 and miR-466 were predicted by miRDB website (http://mirdb.org/). The binding sites between LYAR and miR-466 were predicted by Targetscan website (http://www.targetscan.org/vert_72/). The wild-type of LINC00355 or LYAR that had the predicted miR-466 binding sites was constructed and cloned into a pmir-GLO Dual-luciferase vector (Promega, USA) to obtain the pmirGLO-LINC00355-wild type (LINC00355-WT) or pmirGLO-LYAR-wild type (LYAR-WT) reporter vector. The corresponding mutated sequences were synthesized by GeneTailor Site-Directed Mutagenesis System (Invitrogen) and integrated into the pmir-GLO dual-luciferase vector to form LINC00355-MUT or LYAR-MUT reporter vector. LINC00355-WT, LINC00355-MUT, LYAR-WT, or LYAR-MUT reporter vector were co-transfected with miR-466 mimic or negative control (NC mimic) into NCI-H2170 cells using Lipofectamine 3000. Next, the luciferase activities were determined using the Dual-Glo Luciferase Assay System kit (Promega) after 48 h of transfection, and the data were normalized to renilla luciferase activity.

### RNA binding protein immunoprecipitation (RIP) assay

The interaction between LINC00355 and miR-466 was evaluated by RNA RIP assay using the EZ-Magna RIP kit (Millipore). NCI-H2170 cells were lysed and incubated with magnetic beads conjugated with anti-human Ago2 antibodies or anti-mouse IgG as controls in RIP buffer. Next, the precipitated RNAs were isolated to measure the expression of LINC00355 and miR-466 by qRT-PCR.

### RNA pull-down assay

Full length LINC00355 or mutated LINC00355 was transcribed by T7 RNA polymerase (Stratagene, USA) and labeled with biotin using Biotin RNA labeling mix (Roche, Switzerland). NCI-H2170 cells were lysed using RIPA lysis buffer, followed by incubation with the biotinylated RNA for 2 h at 4°C in RNA RIP buffer (25 mM Tris pH 7.4, 0.5% NP40, 150 mM KCl, 100 mM PMSF, 0.5 mM DTT, and 1× protease inhibitor). Streptavidin agarose beads (Invitrogen) were applied to isolate the complexes, and RNA level was detected by qRT-PCR.

### Reactive oxygen species (ROS) measurement

The level of ROS was evaluated by DCFH-DA (2,7-dichlorodihydrofluorescein diacetate, Beyotime Biotechnology, China) probes. After transfection, NCI-H2170 cells in 24-well plates were cultured with DCFH-DA for 30 min at 37°C. Next, the cells were washed with serum-free DMEM three times and a luminescence spectrometer (480 nm excitation and 530 nm emission; PerkinElmer, USA) was used to obtain intracellular ROS activity.

### Tumor formation assay

BALB/C nude mice (male, 16-18 g, 5-6-week-old) were obtained from Beijing Laboratory Animal Research Center (China) and were maintained in a specific pathogen-free environment with four mice in each cage. NCI-H2170 cells transfected with shLINC00355 or negative control (shNC) were suspended in serum-free DMEM, followed by subcutaneously injected into the right flank of mice. Tumor growth was measured every 7 days and the mice were sacrificed after 35 days.

### Statistical analysis

Each experiment was conducted at least three times. Data were analyzed by SPSS 19.0 software (IBM, USA) and are reported as means±SD. Student's *t*-test was utilized for the comparison between two groups, and one-way ANOVA was used for the comparisons among multiple groups. A P value of less than 0.05 was regarded as a statistically significant difference. The overall survival rate was assessed by Kaplan-Meier method with log-rank test.

## Results

### LINC00355 was over-expressed in lung SCC

**Figure 1:**
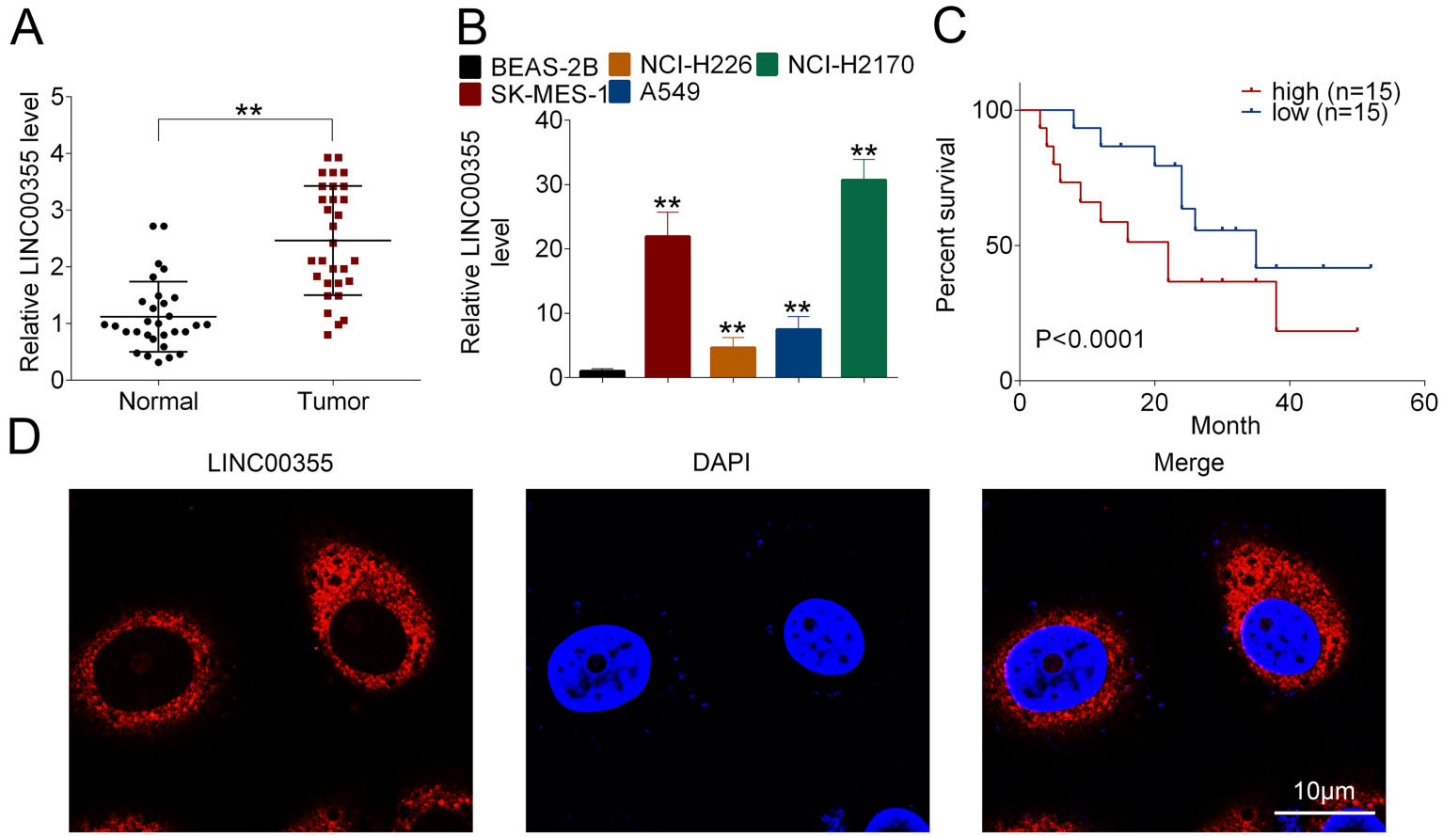
LINC00355 was upregulated in lung squamous cell carcinoma (SCC). The expression of LINC00355 in (**A**) lung SCC tissues and (**B**) cells was determined by qRT-PCR. **C**, The overall survival curves were analyzed using the Kaplan-Meier method followed by log-rank test. **D**, RNA FISH was performed to determine the distribution of LINC00355 in NCI-H2170 cells. Red: LINC00355 was labeled by the RNA probe-LINC00355; blue: cell nuclei were labeled with DAPI. Scale bars=10 μm. Data reported as means±SD. **P<0.01 (ANOVA).

The expression of LINC00355 in 30 pairs of lung SCC specimens (Tumor) and the corresponding adjacent non-tumor (Normal) tissues were determined using qRT-PCR. LINC00355 level was significantly up-regulated in lung SCC tissues compared to the non-tumor tissues (Figure 1A). Moreover, LINC00355 showed higher levels in human lung squamous cell lines (SK-MES-1, NCI-H226, A549, and NCI-H2170) than in human normal lung epithelial cell line BEAS-2B (Figure 1B). Among them, NCI-H2170 cells showed the highest expression level of LINC00355 compared to other human lung SCC lines, which was thus chosen for the following experiments.

Patients with lung SCC were divided into two groups (high LINC00355 expression group and low LINC00355 expression group) according to the median expression level of LINC00355. Figure 1C shows that the overall survival rate of patients with the low LINC00355 expression was significantly higher than that with high LINC00355 expression indicating that LINC00355 expression level predicted poor prognosis of lung SCC patients.

In addition, the distribution of LINC00355 in lung SCC cells was detected using RNA FISH. Figure 1D indicates that LINC00355 was mainly located in the cytoplasm of NCI-H2170 cells.

### LINC00355 regulated lung SCC cell proliferation, apoptosis, migration, and invasion

**Figure 2:**
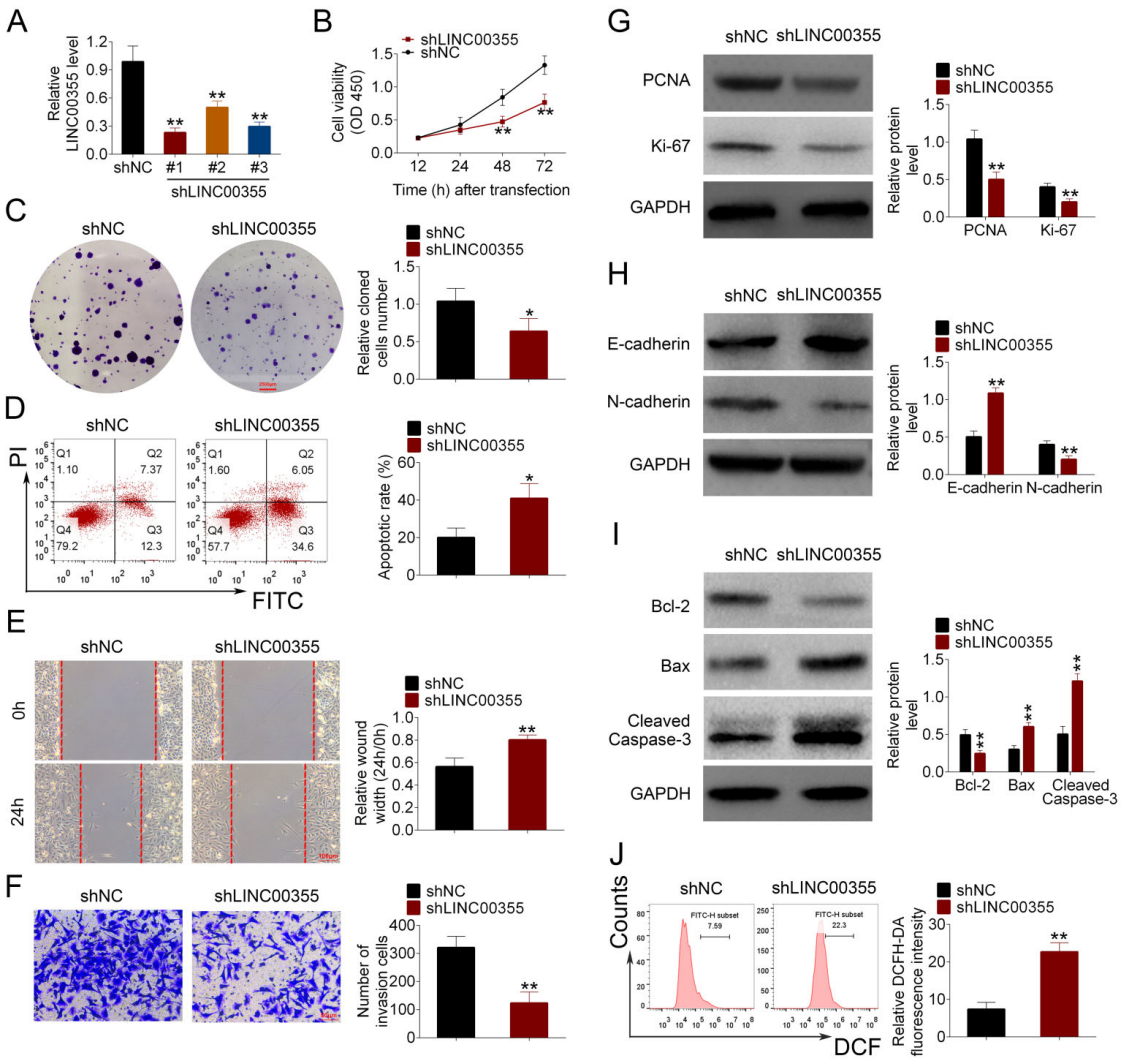
LINC00355 regulated lung squamous cell carcinoma (SCC) cell proliferation, apoptosis, migration, and invasion. **A**, qRT-PCR was used to measure the expression of LINC00355. Cell proliferation was detected by (**B**) CCK-8 assay and (**C**) colony formation assay (scale bar=2500 μm). **D**, Cell apoptosis was examined by flow cytometry. Cell migration and invasion were determined by (**E**) wound healing assay (scale bar=100 μm) and (**F**) transwell invasion assay (scale bar=50 μm). The protein expression levels of (**G**) PCNA, (**H**) E-cadherin, N-cadherin, and (**I**) Bcl-2, BAX, and cleaved caspase-3 were measured by western blot. **J**, Reactive oxygen species (ROS) were evaluated using DCFH-DA probes. Data reported as means±SD. *P<0.05, **P<0.01 compared to shNC (negative control) (Student's *t*-test).

To detect the effects of LINC00355 on lung SCC cells, the proliferation, apoptosis, migration, and invasion of NCI-H2170 cells transfected with LINC00355 shRNA were determined. After NCI-H2170 cells were transfected with shRNAs of LINC00355 (#1 shLINC00355, #2 shLINC00355, #3 shLINC00355), the transfection efficiencies of shLINC00355 were determined (Figure 2A). Among them, #1 showed the lowest expression level of LINC00355 compared to #2 and #3, which was chosen for the subsequent experiments.

CCK8 assay revealed that the viability of NCI-H2170 cells was considerably inhibited by sh-LINC00355 compared to the shNC group (Figure 2B). LINC00355 knockdown also significantly suppressed the colony formation of NCI-H2170 cells compared to the shNC group (Figure 2C). Moreover, the protein level of PCNA (proliferating cell nuclear antigen) was greatly down-regulated in NCI-H2170 cells by transfection of shLINC00355 compared to the shNC group (Figure 2G).

The effect of LINC00355 on NCI-H2170 cell apoptosis was evaluated by flow cytometry and western blot. Figure 2D demonstrates that the apoptotic rate of NCI-H2170 cells in the shLINC00355 group was significantly higher than that in the shNC group. Western blot further confirmed that knockdown of LINC00355 considerably reduced the expression level of the anti-apoptotic protein (Bcl-2) and enhanced the levels of pro-apoptotic proteins (Bax and cleaved caspase-3) in NCI-H2170 cells compared to the shNC group (Figure 2I). Thus, LINC00355 knockdown significantly promoted the apoptosis of NCI-H2170 cells.

The effects of LINC00355 on NCI-H2170 cell migration and invasion were respectively assessed by wound healing and transwell invasion assays. Compared to the control group, knockdown of LINC00355 greatly suppressed the migration of NCI-H2170 cells and decreased the number of invasive NCI-H2170 cells (Figure 2E and F). Figure 2H shows that knockdown of LINC00355 increased the expression level of E-cadherin but decreased the level of N-cadherin compared to the shNC group. Based on the fact that low expression of E-cadherin and high level of N-cadherin can contribute to cell migration and invasion (22), it can be concluded that knockdown of LINC00355 inhibited the migration and invasion of NCI-H2170 cells.

Furthermore, compared to the shNC group, the higher fluorescence intensity in the shLINC00355 group suggested that knockdown of LINC00355 caused the excessive generation of ROS (Figure 2J).

### LINC00355 targeted miR-466 as competing endogenous RNA in lung SCC cells

**Figure 3:**
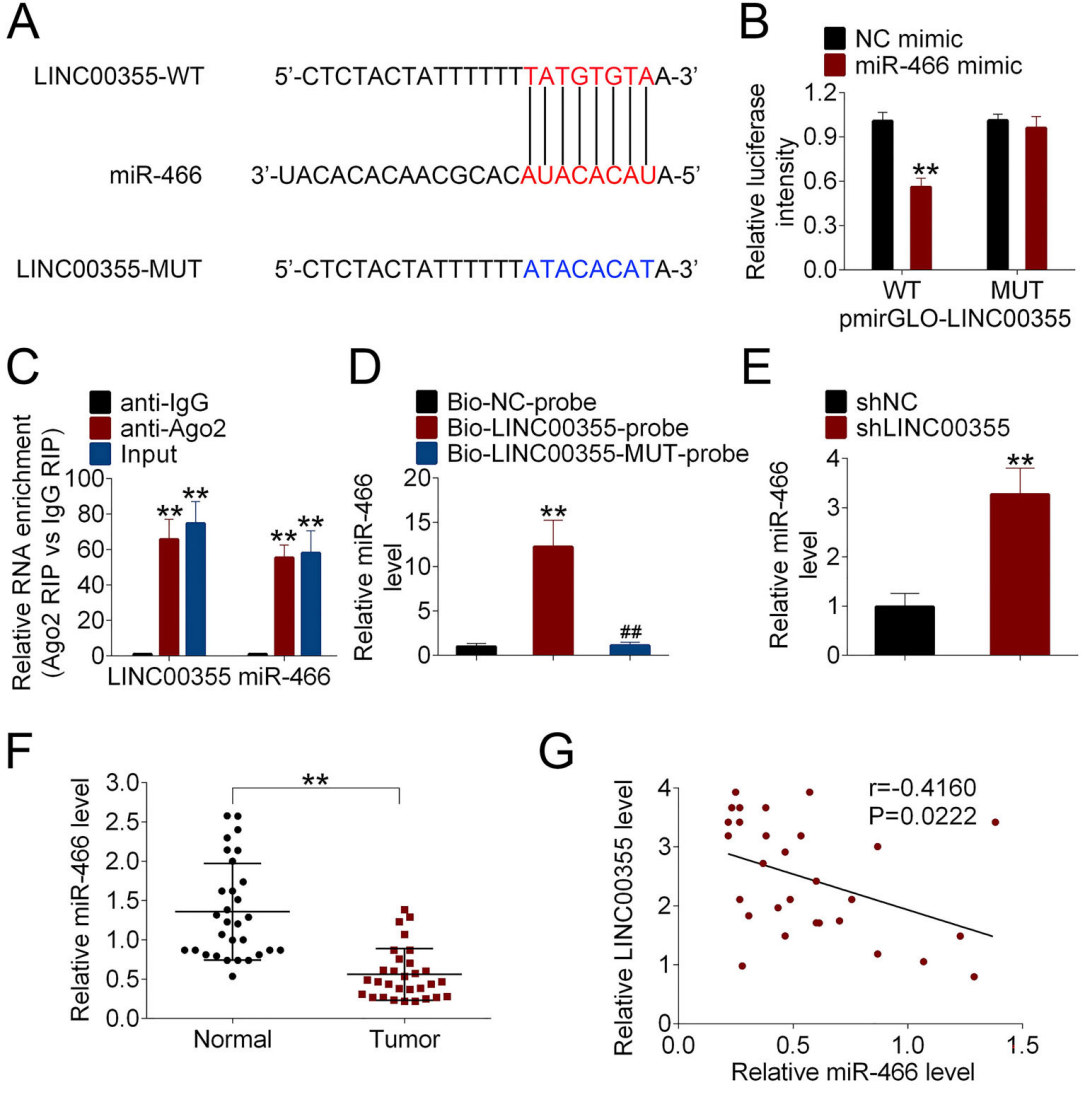
LINC00355 directly interacted with miR-466. **A**, The putative binding sites between LINC00355 and miR-466 were predicted by the miRDB website. The interaction between LINC00355 and miR-466 was determined by (**B**) luciferase reporter assay, (**C**) RIP, and (**D**) RNA pull-down assays. **E**, The expression of miR-466 was detected by qRT-PCR. **F**, The expression of miR-466 in lung squamous cell carcinoma tissues and the corresponding adjacent normal tissues was measured by qRT-PCR. **G**, The correlation of LINC00355 with miR-466 was evaluated by Pearson's linear regression analysis. **P<0.01 compared to the respective controls (NC, anti-IgG). Data reported as means±SD. ^##^P<0.01 compared to Bio-LINC00355-probe (Student's *t*-test or ANOVA). WT: wild type; MUT: mutated; NC: negative control.

The miRDB website was applied to identify the potential target miRNAs of LINC00355, and miR-466 was chosen as a representative miRNA; the putative binding sites are listed in Figure 3A. The luciferase activity was decreased after NCI-H2170 cells were co-transfected with LINC00355-WT reporter vector and miR-466 mimic compared with the negative control group, while luciferase activity showed no significant difference between LINC00355-MUT+miR-466 mimic and LINC00355-MUT+NC mimic groups (Figure 3B). The interaction between LINC00355 and miR-466 was further validated by RIP and RNA pull-down assays. Compared to the anti-lgG group, both LINC00355 and miR-466 were enriched by anti-Ago2 (Figure 3C). Figure 3D shows a significant amount of miR-466 in the LINC00355 pulled down pellet compared with the control group. Collectively, LINC00355 could directly interact with miR-466.

The level of miR-466 was significantly up-regulated after NCI-H2170 cells were transfected with shLINC00355 compared to the control group (Figure 3E). In addition, the level of miR-466 in lung SCC tissues was significantly lower than that in the corresponding adjacent normal tissues (Figure 3F). A linear regression analysis found a negative correlation between LINC00355 expression and miR-466 level (Figure 3G).

### LYAR was a direct target of miR-466

**Figure 4:**
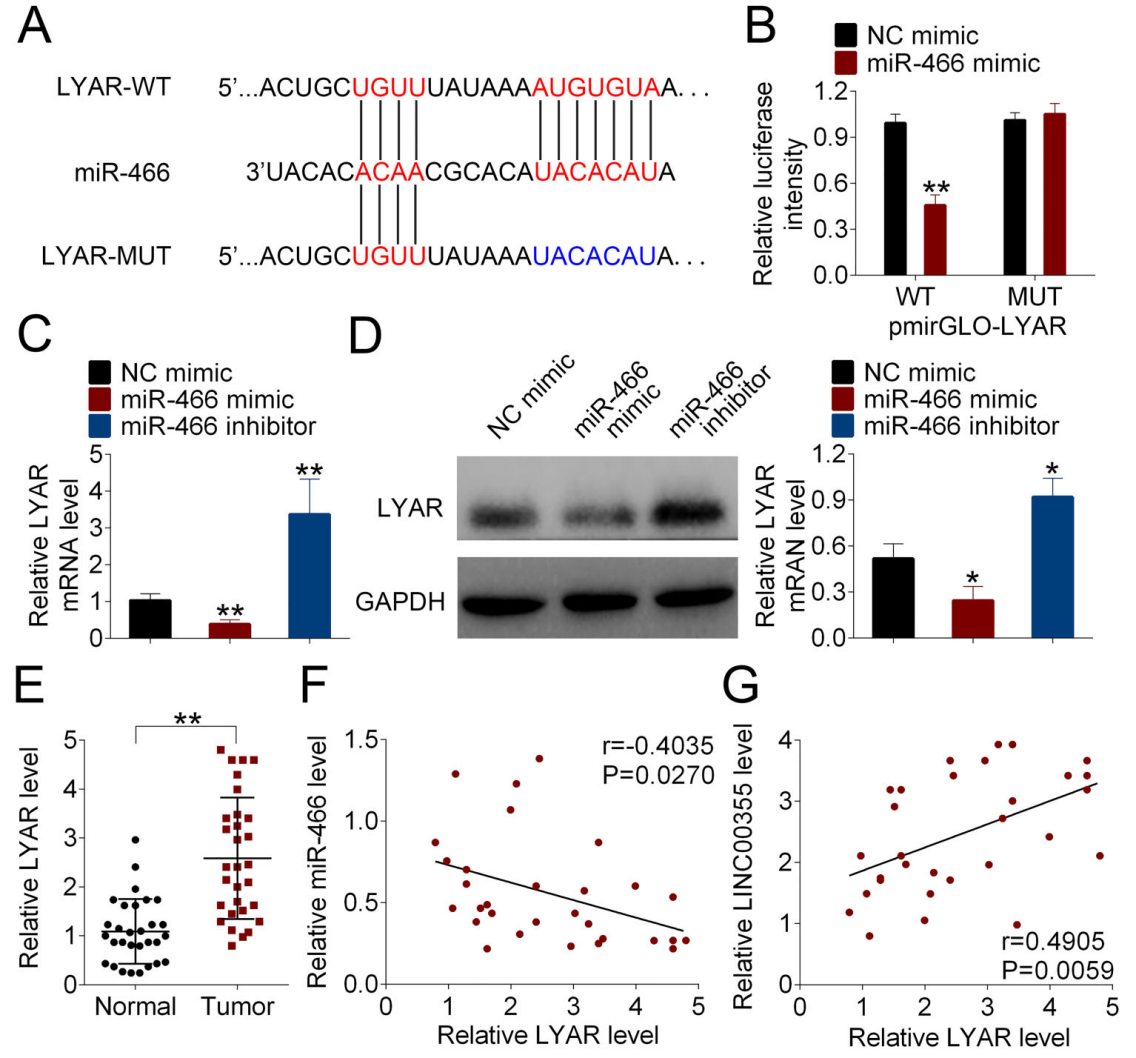
LYAR was a direct target of miR-466. **A**, The putative binding sites between miR-466 and LYAR were predicted by Targetscan website. **B**, The interaction between miR-466 and LYAR was validated by luciferase reporter assay. **C**, The mRNA and (**D**) protein expression levels of LYAR were respectively measured by qRT-PCR and western blot. **E**, The expression of LYAR in lung SCC tissues and corresponding adjacent normal tissues was examined by qRT-PCR. **F** and **G**, The correlation between LYAR and miR-466 or LINC00355 was assessed by Pearson's linear regression analysis. Data are reported as means±SD. *P<0.05, **P<0.01 compared to NC (Student's *t*-test or ANOVA). WT: wild type; MUT: mutated; NC: negative control.

Figure 4A reveals the putative binding sites between LYAR and miR-466. The luciferase activity of LYAR-WT was significantly suppressed after co-transfection with miR-466 mimic in NCI-H2170 cells compared to the control group, and yet the activity of LYAR-MUT had no significant difference between the miR-466 mimic and mimic control groups (Figure 4B). These results suggested that LYAR could directly target miR-466.

After miR-466 mimic, miR-466 inhibitor, or negative control mimic (NC mimic) was transfected into NCI-H2170 cells, the level of LYAR was decreased by miR-466 mimic transfection, but increased by miR-466 inhibitor compared to the control group (Figure 4C). Compared to the control group, western blot further confirmed that miR-466 mimics decreased the protein expression of LYAR, and miR-466 inhibition caused the opposite results (Figure 4D). Additionally, the aberrantly higher expression of LYAR in lung SCC tissues was also observed compared to the corresponding normal tissues (Figure 4E). Pearson's linear regression analysis indicated that LYAR expression was negatively correlated with miR-466 level but positively correlated with LINC00355 level (Figure 4F and G).

### LINC00355 regulated lung SCC cell proliferation, apoptosis, migration, and invasion through mediating the miR-466/LYAR axis

**Figure 5:**
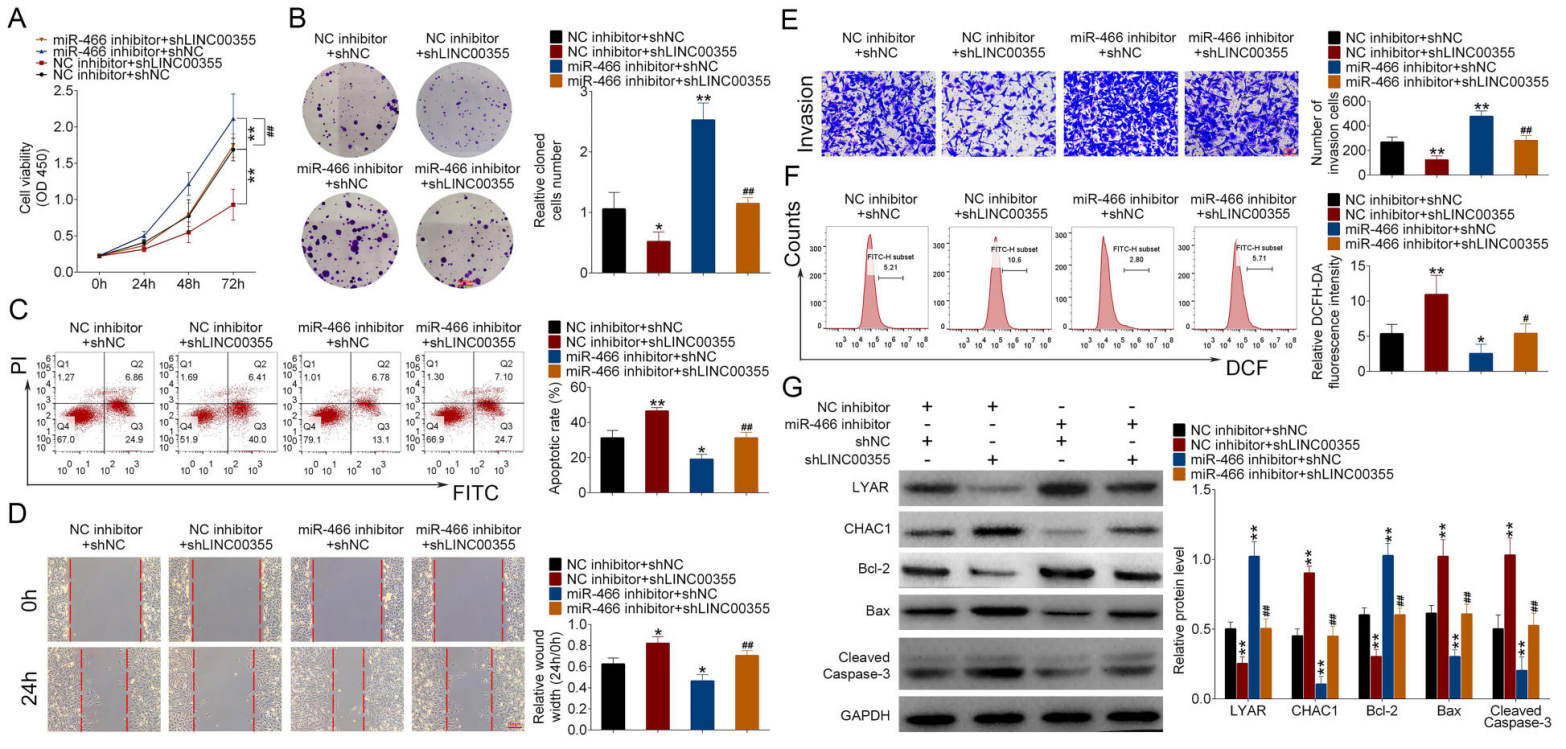
LINC00355/miR-466/LYAR regulated the proliferation, apoptosis, migration, and invasion of lung squamous cell carcinoma cells. Cell proliferation was measured by (**A**) CCK-8 and (**B**) colony formation assays (scale bar=2500 μm). **C**, Cell apoptosis was assessed using flow cytometry. Cell migration and invasion were detected by (**D**) wound healing assay (scale bar=100 μm) and (**E**) transwell invasion assay (scale bar=50 μm). **F**, Reactive oxygen species were evaluated using DCFH-DA probes. **G**, Protein expression levels of LYAR, CHAC1, Bcl-2, BAX, and cleaved caspase-3 were determined by western blot. Data reported as means±SD. *P<0.05, **P<0.01 compared to the respective control. ^#^P<0.05, ^##^P<0.01 compared to NC inhibitor + shNC LINC00355 (ANOVA). NC: negative control.

Further analysis was performed to investigate whether miR-466/LYAR axis was involved in the effects of LINC00355 on lung SCC cell growth. Compared to the NC inhibitor + shNC group, knockdown of LINC00355 inhibited cell viability and colony formation (Figure 5A and B). However, co-transfection of shLINC00355 and miR-466 inhibitor in NCI-H2170 cells reversed these effects compared to the NC inhibitor + shNC LINC00355 group. Compared with the NC inhibitor + shNC group, the apoptotic rate of NCI-H2170 cells was decreased by shLINC00355 transfection, which was greatly reversed by co-transfection of shLINC00355 and miR-466 inhibitor (Figure 5C). Moreover, the up-regulation of pro-apoptotic proteins (BAX and cleaved caspase-3) and the down-regulation of the anti-apoptotic protein Bcl-2 in the NC inhibitor + shNC LINC00355 group were also reversed by co-transfection of shLINC00355 and miR-466 inhibitor in NCI-H2170 cells (Figure 5G). The inhibited migration and invasion of NCI-H2170 cells in the NC inhibitor + shNC LINC00355 group were reversed by co-transfection of shLINC00355 and miR-466 inhibitor (Figure 5D and E). Meanwhile, the excessive generation of ROS in the NC inhibitor + shNC LINC00355 group was reversed by co-transfection of shLINC00355 and miR-466 inhibitor in NCI-H2170 cells (Figure 5F). Western blot showed that the expression of CHAC1 was significantly increased in the NC inhibitor + shNC LINC00355 group, which was reversed by co-transfection of shLINC00355 and miR-466 inhibitor in NCI-H2170 cells (Figure 5G). Additionally, the decreased expression of LYAR in the NC inhibitor + shNC LINC00355 group was reversed by co-transfection of shLINC00355 and miR-466 inhibitor in NCI-H2170 cells (Figure 5G). Therefore, LINC00355 could regulate lung SCC cell proliferation, apoptosis, migration, and invasion through mediating the miR-466/LYAR axis.

### Knockdown of LINC00355 inhibited tumor growth in lung SCC

**Figure 6:**
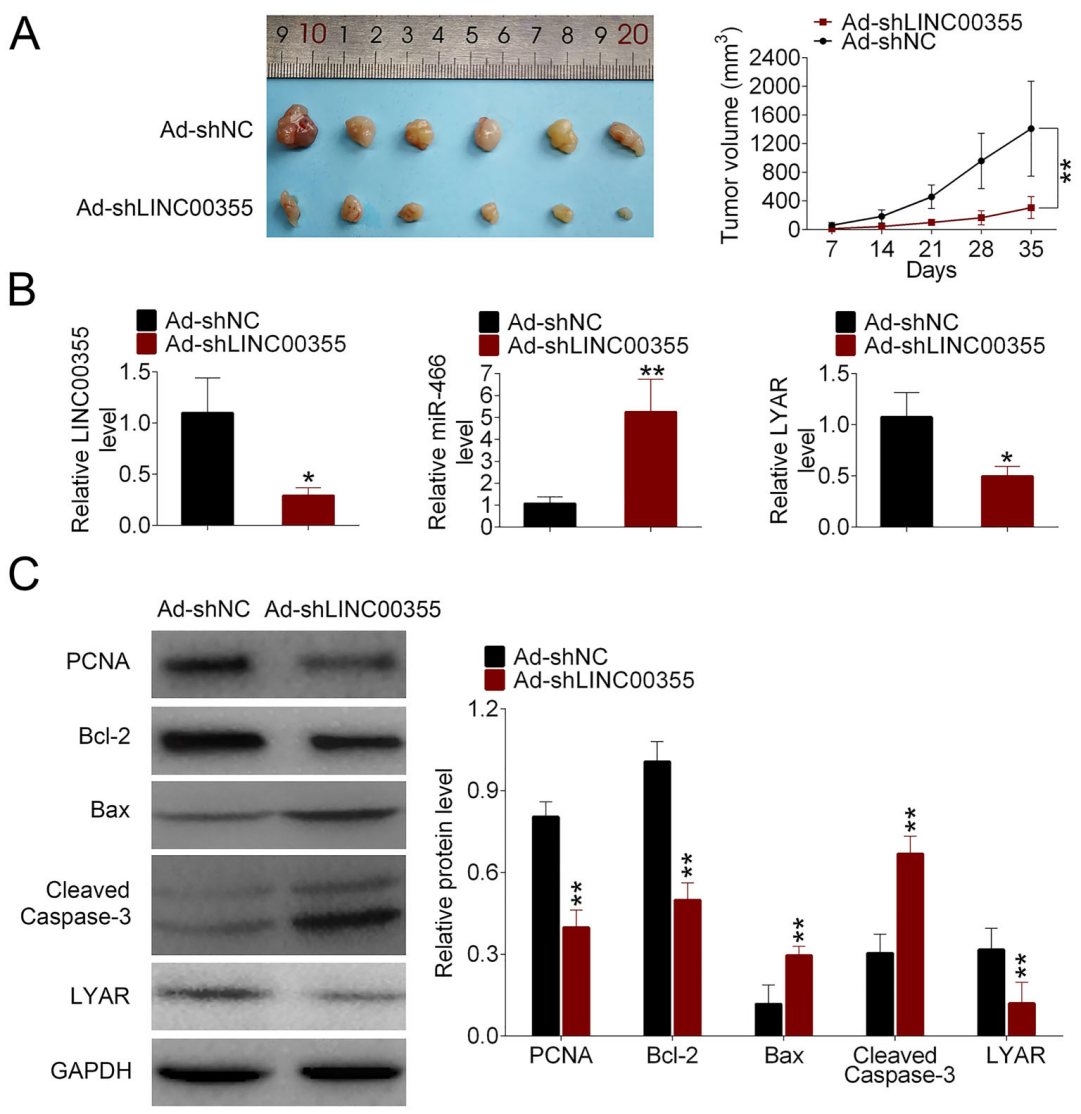
Knockdown of LINC00355 inhibited tumor growth in lung squamous cell carcinoma. **A**, After 35 days, BALB/C nude mice were sacrificed, and the representative images of lung SCC tumors are shown. **B**, The expression of LINC00355, miR-466, and LYAR was evaluated by qRT-PCR. **C**, Protein expression levels of PCNA, Bcl-2, BAX, cleaved caspase-3, and LYAR were measured by western blot. Data reported as means±SD. *P<0.05, **P<0.01 compared to control (NC) (Student's *t*-test).

As shown in Figure 6A, the size and volume of the tumors were significantly suppressed by LINC00355 knockdown compared to the control group. The higher level of miR-466 and lower expression of LINC00355 and LYAR were observed in the Ad-shLINC00355 group compared to the Ad-shNC group using qRT-PCR (Figure 6B). Consistently, the lower protein expression of LYAR in the Ad-shLINC00355 group compared to the Ad-shNC group was confirmed by western blot (Figure 6C). In addition, the protein level of PCNA was decreased in the Ad-shLINC00355 group compared to the Ad-shNC group. Meanwhile, LINC00355 knockdown increased the protein levels of Bax and cleaved caspase-3 and decreased the level of Bcl-2 compared to the Ad-shNC group.

## Discussion

In our study, LINC00355 was upregulated in lung SCC tissues and cells, which was positively correlated with poor prognosis in lung SCC patients. LINC00355 was mainly located in the cytoplasm of NCI-H2170 cells. In addition, LINC0035 expression negatively correlated with miR-466 level, and positively correlated with LYAR level. Knockdown of LINC00355 inhibited proliferation, migration, and invasion and promoted apoptosis of NCI-H2170 cells through regulating the miR-466/LYAR axis. Moreover, knockdown of LINC00355 decreased LYAR expression to suppress the tumor growth in lung SCC via mediating miR-466 expression.

Effective treatment options to improve overall survival in patients with lung SCC are insufficient (4,5). Hence, exploring the molecular mechanisms underlying the pathogenesis of lung SCC is a hot topic (7). lncRNAs have gradually attracted more and more attention due to their specific biological functions in various cancers (10). LINC00355, as a newly identified lncRNA, has been found to be relevant with the overall survival of human colon adenocarcinoma (18). There is no literature regarding the effect of LINC00355 on lung SCC. To solve this issue, a series of biological experiments were performed both *in vitro* and *in vivo*.

These investigations indicated that LINC00355 knockdown suppressed SCC cell growth. ROS are associated with various signal transduction events, and thereby regulate cell proliferation, apoptosis, migration, invasion, and differentiation (23-26). In this study, compared to the shNC group, the higher fluorescence intensity in the shLINC00355 group suggested that knockdown of LINC00355 caused the excessive generation of ROS. Thus, knockdown of LINC00355 could increase the level of ROS to influence the proliferation, apoptosis, migration, and invasion of NCI-H2170 cells. CHAC1, an oxidative stress gene, has been shown to considerably induce oxidative stress (27). Here, western blot showed that the expression of CHAC1 was significantly increased in the NC inhibitor + shNC LINC00355 group compared to the NC inhibitor + shNC group, indicating that LINC00355 could induce oxidative stress. As far as we know, this is the first evidence demonstrating the role of LINC00355 in regulating oxidative stress. Taken together, LINC00355 could be considered a potential biomarker for the diagnosis and treatment of lung SCC.

It has been reported that LINC00355 interacts with chromatin and regulates the transcription and processing of RNA (14). To explore the molecular mechanism of LINC00355 on lung SCC, the ceRNA hypothesis has been utilized in the present study. The ceRNA hypothesis suggests that specific lncRNA can function as a sponge to impair miRNA activity, thus indirectly upregulate miRNA target gene expression (28,29). For instance, lncRNA ATB can function as a ceRNA to promote the development of esophageal SCC through regulating the miR-200b/Kindlin-2 axis (30). Here, miRDB website predicted miR-466 as a representative target miRNA of LINC00355. The subsequent experiments including luciferase reporter assay, RIP, and RNA pull-down assays showed that LINC00355 could directly target miR-466 and negatively regulate its expression.

miR-466 has been shown to be a tumor suppressor in osteosarcoma (31) and colorectal cancer (32). miR-466 regulates its target gene RUNX2 to inhibit bone metastasis and tumor growth in prostate cancer (33).

Targetscan website predicted LYAR as a representative target gene of miR-466. In addition, LYAR negatively correlated with miR-466 and was positively regulated by LINC00355. LYAR is known as a nucleolar zinc finger protein that is associated with ribosome processing, self-renewal of embryonic stem cells, and cell growth (34-36). LYAR has been regarded as a nucleolar onco-protein to regulate cell growth (37). Moreover, LYAR overexpression promotes cell proliferation and survival in neuroblastoma (38). Consistently, our study found that LYAR was over-expressed in lung SCC tissues and participated in the regulation of NCI-H2170 in lung SCC cell growth.

The interaction among LINC00355, miR-466, and LYAR in lung SCC cells and tumors was further explored. The results showed that miR-466 inhibitor reversed the effects of LINC00355 knockdown on lung SCC cell growth, suggesting that LINC00355 knockdown could inhibit proliferation, migration, and invasion, but promote apoptosis of lung SCC cells through regulating the miR-466/LYAR axis. Taken together, LINC00355 knockdown suppressed the growth of lung SCC tumors via targeting miR-466 to down-regulate LYAR expression. A previous study indicated that LINC00355 competitively binds to miR-195, resulting in the upregulation of HOXA10, thus promoting the progression of head and neck squamous cell carcinoma (39).

In conclusion, our results provide a new sight for understanding the molecular mechanism of lung SCC, and offers potential biomarkers for the diagnosis and treatment of this disease.

## Supplementary Material

Click here to view [pdf].
